# Concave Microwell Formation Induced by PDMS Water Vapor Permeability for Spheroid Generation

**DOI:** 10.3390/mi15121496

**Published:** 2024-12-14

**Authors:** Min-Cheol Lim, Tai-Yong Kim, Gyeongsik Ok, Hyun Jung Kim, Yun-Sang Choi, Young-Rok Kim

**Affiliations:** 1 Research Group of Food Safety and Distribution, Korea Food Research Institute (KFRI), Wanju 55365, Republic of Korea; 2 Department of Food Biotechnology, Korea University of Science and Technology, Daejeon 34113, Republic of Korea; 3 Research Group of Food Processing, Korea Food Research Institute (KFRI), Wanju 55365, Republic of Korea; 4 Institute of Life Science and Resources & Department of Food Science and Biotechnology, College of Life Sciences, Kyung Hee University, Yongin 17104, Republic of Korea

**Keywords:** concave microwells, PDMS vapor permeability, spheroid formation, water vapor evaporation, 3D cell culture

## Abstract

This study introduces a novel method for the fabrication of concave microwells involving water vapor permeation through polydimethylsiloxane (PDMS). This method leverages the exceptional water vapor permeability of PDMS to enable a scalable and cost-effective fabrication process, addressing the limitations of existing techniques such as photolithography that are resource-intensive and complex. PDMS is more permeable to water vapor than to other gas molecules, resulting in the formation of microwells. Smooth-sloped concave microwells are formed by depositing droplets of 10% ethylene glycol on a PDMS substrate followed by curing at 70 °C and evaporation of water vapor. These microwells exhibit a unique structural gradient that is highly conducive for biological applications. Concave microwells were further used as a platform to generate animal cell spheroids, demonstrating their potential for three-dimensional cell culture. Unlike conventional methods, this approach allows precise control over microwell morphology by simply adjusting droplet size and curing conditions, offering enhanced tunability and reproducibility. The formation yield of these microwells is dependent on the volume of the water droplets, demonstrating the importance of droplet size in controlling microwell morphology. This approach provides a simple and effective method for creating microwells without complex lithographic processes, making it a highly promising tool for a range of biomedical applications, including tissue engineering, cancer research, and high-throughput drug screening.

## 1. Introduction

Three-dimensional (3D) cell culture systems provide physiologically relevant environments that more closely resemble in vivo conditions than traditional two-dimensional (2D) cell culture methods and have thus revolutionized biomedical research [1]. Among numerous 3D culture techniques, microwells have proved an indispensable tool that facilitates cell aggregation and enables the formation of multicellular spheroids. These confined microenvironments facilitate research in various fields, such as tissue engineering, drug discovery, and cancer biology, by replicating structural and functional characteristics of natural tissues. In particular, spheroids formed in microwells show considerable promise in these applications owing to their ability to model complex in vivo cell–cell interactions and microenvironments [2]. While spheroid generation techniques such as hanging drop methods, spinner flask bioreactors, and hydrogel scaffolds have been widely employed, these approaches often encounter challenges such as operational complexity, limited reproducibility, and difficulties in scaling for high-throughput applications.

Polydimethylsiloxane (PDMS) is commonly used in the fabrication of microwells, owing to its high biocompatibility, optical transparency, and ease of handling [3]. Although traditional microwell fabrication techniques, including photolithography and soft lithography, afford microwells with precise geometries, these methods are often resource-intensive and require specialized equipment and expertise, which limits their accessibility and scalability [4]. Moreover, the formation of smooth-sloped concave microwells, which are ideal for spheroid culture, using conventional techniques presents a considerable challenge. Accordingly, the development of simple, cost-effective, and scalable alternatives for widespread use has attracted growing interest [5].

PDMS is distinguished by its high permeability to gases, particularly water vapor, which presents an opportunity for innovative microwell fabrication [6]. The deposition of droplets of aqueous solutions such as ethylene glycol (EG) on PDMS and subsequent selective evaporation of water through the PDMS matrix induces the formation of concave microwells [7]. This novel method bypasses the requirement for lithographic techniques by leveraging the inherent properties of PDMS, thereby simplifying the microwell fabrication process. Additionally, the dimensions and yield of microwells can be precisely controlled by adjusting the droplet volume, providing a versatile platform for the formation of microwells with tailored geometries [8].

In this study, we present a novel approach for the fabrication of smooth-sloped concave microwells that exploits the water vapor permeation properties of PDMS. The fabricated microwells were validated as platforms for 3D cell culture, specifically for the formation of SW1222 cell spheroids, demonstrating their utility in tissue engineering, drug testing, and cancer research [9]. Furthermore, this microwell platform can support the aggregation and differentiation of muscle cells to replicate the structure and texture of natural tissues and thus has potential applications in the cultured meat industry. This study highlights the simplicity and versatility of microwell fabrication using PDMS and provides a valuable tool that is expected to facilitate advances in biomedical research and biotechnology.

## 2. Materials and Methods

### 2.1. Materials

Elastomer PDMS Sylgard 184, consisting of a monomer and curing agent, was purchased from Dow Corning (Midland, MI, USA). Ethyl alcohol (EG), Dulbecco’s modified Eagle’s medium (DMEM), fetal bovine serum (FBS), and penicillin–streptomycin were purchased from Sigma-Aldrich (St. Louis, MO, USA). SU-8 2100 photoresist was purchased from MicroChem Corp. (Newton, MA, USA). The human colon adenocarcinoma SW1222 cell line was acquired from ECACC (Salisbury, UK).

### 2.2. Preparation of Uncured PDMS Solution

PDMS solutions were prepared by mixing the PDMS base with a curing agent at ratios of 5:1, 10:1, 20:1, and 40:1. The solutions were thoroughly degassed in a vacuum desiccator to remove air bubbles immediately prior to use. The elasticity and gas permeability of the cross-linked PDMS substrate can be adjusted based on the mixing ratio of the base and curing agent. Thus, subsequent microwell fabrication was performed using an appropriate mixing ratio.

### 2.3. Microwell Fabrication via Water Vapor Permeation

**Scheme 1:**
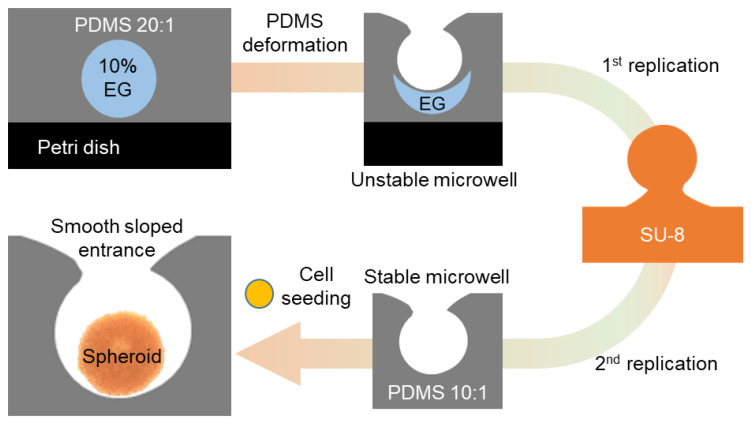
Schematic representation of microwell fabrication, replication, and spheroid formation using PDMS and SU-8.

Droplets of a 10% EG solution were deposited on the liquid PDMS solution [10]. To fabricate the microwell structure, the PDMS solution (20 g) was added to a petri dish with a diameter of 8.5 cm, and a 2 µL of 10% EG solution was applied onto the leveled surface of the PDMS before the mixture was cured in a dry oven at 70 °C for 24 h. To determine the PDMS mixture ratio at which the surface of the PDMS substrate contracted owing to the EG droplets, an EG solution of the same concentration and volume was applied to PDMS substrates prepared at ratios of 5:1, 10:1, 20:1, and 40:1. Curing was then performed at 70 °C for 12 h, and the condition with the highest curing agent ratio that induced deformation of the PDMS surface was selected for subsequent experiments. Scheme 1 illustrates the overall process of microwell fabrication, replication, and spheroid formation. The initial step involves the creation of unstable concave microwells on a PDMS (20:1) substrate using 10% EG droplets, followed by replication with SU-8 to ensure structural stability. A second replication using PDMS (10:1) produces stable microwells with smooth-sloped entrances, which were subsequently utilized for cell seeding and spheroid formation experiments.

### 2.4. Fabrication of SU-8 Molds for Microwell Replication

To ensure the structural stability of the PDMS microwell arrays, a mold with the inverse profile of the microwells was fabricated using SU-8, a polymer with high mechanical strength [11]. The SU-8 solution was applied to the surface of the PDMS microwells, and polymerization was initiated using 365 nm UV light from a handheld UV lamp for 30 min to cure SU-8. Once fully solidified, the SU-8 mold was detached from the PDMS substrate, affording a durable negative mold for the subsequent replication processes. To further adjust the dimensions of the SU-8 mold, the volume of the EG solution was varied to 0.5, 1, and 2 µL under otherwise identical conditions. The SU-8 solution was used to replicate the concave PDMS microwell structures by UV curing in accordance with an established method, producing mushroom-shaped micropillar molds that served as negative templates, enabling precise replication of the microwell arrays in subsequent experiments.

### 2.5. Replication of Microwells Using 10:1 PDMS

To replicate the microwell structures, a 10:1 PDMS mixture of base and curing agent was prepared, thoroughly degassed, and poured over the prefabricated SU-8 molds in petri dishes. The PDMS layer was then cured at 70 °C for 24 h to ensure complete solidification before being removed from the SU-8 mold, yielding high-fidelity replicas of the original microwell design with precisely defined smooth-sloped concave structures. The replicated PDMS microwell substrates were rinsed consecutively with distilled water, filtered water, and pure ethanol to remove any potential surface residue and stored in sterilized containers in a desiccator to maintain sterility and prevent contamination. The microwell structures were preserved under these conditions prior to further experiments.

### 2.6. Cell Culture and Spheroid Formation

SW1222 human colon adenocarcinoma cells were cultured in DMEM supplemented with 10% FBS and 1% penicillin–streptomycin to maintain optimal cell proliferation conditions (37 °C, 5% CO₂). All cell culture procedures adhered to institutional guidelines and were approved under the relevant ethical regulations for in vitro experiments. The SW1222 cell line was obtained with proper permissions and in compliance with regulatory standards. PDMS microwell arrays fabricated by water vapor permeation served as scaffolds for spheroid formation. Prior to cell seeding, the microwell surfaces were sterilized with ethyl alcohol, rinsed with phosphate-buffered saline to eliminate residual ethanol, and then prepared for cell culture. Following sterilization, the SW1222 cells were suspended in DMEM (1 × 10^5^ cells/mL). A defined volume of cell suspension was added to the PDMS microwell arrays to ensure an even distribution of cells across the concave microwells. The microwell plates were incubated at 37 °C in a humidified atmosphere containing 5% CO₂, allowing the cells to settle and attach to the microwells. The cells aggregated naturally at the bottom of the concave microwells, promoting three-dimensional cell-to-cell interactions that facilitate spheroid formation. After an initial attachment period of 24 h, the medium was refreshed to remove nonadherent cells and provide nutrients for spheroid maturation. Incubation was continued for 3 days, during which time the medium was changed daily to maintain optimal culture conditions and promote uniform spheroid growth. Observations were performed daily under a microscope to monitor spheroid formation, size, and integrity.

### 2.7. Microstructural Analysis

The morphology and dimensions of the microwells and cell spheroids were evaluated using optical microscopy and scanning electron microscopy (SEM) using an inverted microscope (Nikon Eclipse TE2000U, Nikon, Melville, NY, USA) and TM-3000 scanning electron microscope (Hitachi, Tokyo, Japan), respectively. Images were captured to monitor spheroid formation, and the surface and structure of the microwells was analyzed at high resolution using SEM.

## 3. Results and Discussion

### 3.1. Concave Microwell Fabrication via Water Vapor Permeation Through PDMS Membrane

The unique gas permeability of PDMS, particularly its high permeability to water vapor compared to other gases such as oxygen and nitrogen [12], were exploited to fabricate the concave microwells without lithography. The exceptional permeability of water vapor through PDMS, with a rate of 3600 × 10⁻⁹ cm³·cm/(cm²·s·cmHg), is approximately 60 times that of oxygen (60 × 10⁻⁹ cm³·cm/(cm²·s·cmHg)) and 125 times that of nitrogen (28 × 10⁻⁹ cm³·cm/(cm²·s·cmHg)) [12]. The permeability of carbon dioxide through PDMS is also very high, at 325 × 10⁻⁹ cm³·cm/(cm²·s·cmHg), yet this is still an order of magnitude lower than that of water vapor [12]. This stark contrast underscores the anomalously high water vapor permeability, particularly considering the low solubility of water in PDMS (less than 1 ppm), which is far lower than that of CO₂ and other gases that rely on solubility-driven transport [13].

The formation of microwells is driven by the water vapor-selective permeability of PDMS, which enables the controlled evaporation of water from droplets deposited on its surface, forming concave microwell structures without the need for external forces or complex fabrication processes. The high water vapor permeability of PDMS is attributed to its unique molecular properties, including long Si–O and Si–C bonds, high fractional free volume, and low glass transition temperature, which result in the formation of dynamic sub-nanometer hydrophobic pores. These nanopores facilitate the selective transport of water vapor from regions of high to low vapor pressure through hydrogen-bonded chains of water molecules. Unlike the solubility-driven transport of other permeable gases, water transport is driven by the structural flexibility and compressibility of PDMS, which operates similarly to the transport mechanisms observed in carbon nanotubes and aquaporins [13].

**Figure 1:**
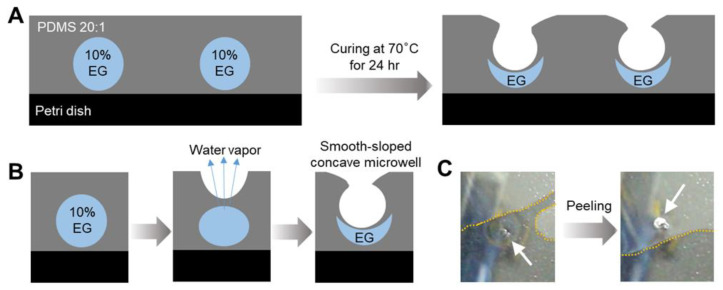
Fabrication of smooth-sloped concave microwells using water vapor evaporation. (**A**) Formation of concave microwells on a PDMS (20:1) substrate through curing at 70 °C for 24 h. 10% EG droplets are initially deposited on the PDMS surface. As the system cures, the structure of the microwell takes shape. (**B**) Schematic of water vapor evaporation from the EG droplets to form smooth-sloped concave microwells. (**C**) Optical image of the smooth-sloped concave microwells. The dotted yellow line outlines the edges of the microwell structure. The peeling process reveals the smooth concave morphology of the structure.

**Figure 2:**
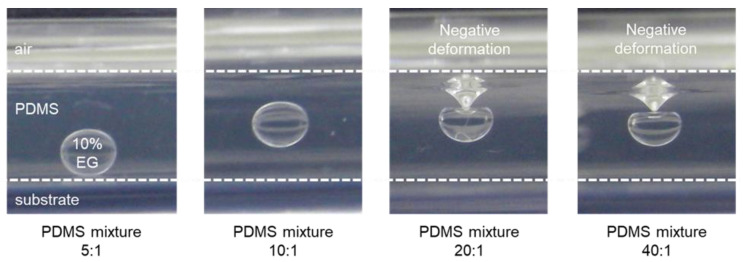
Effect of elastic properties of PDMS on droplet-induced negative deformation.

Droplets containing a mixture of EG and water were deposited onto a PDMS substrate (Figure 1). During curing of the PDMS at 70 °C, the water evaporates through the PDMS membrane owing to its high water vapor permeability, while the EG remains trapped. This process eventually forms concave microwells. The selective permeation of water vapor through the PDMS surface is the driving mechanism for the formation of the microwells, enabling precise control over the morphology of the structure by changing the droplet size and composition. Both the microwell formation process and the mechanical properties of the substrate are influenced by the PDMS-to-curing agent ratio [14]. Substrates derived from PDMS mixtures with higher curing agent ratios (e.g., 5:1 or 10:1) exhibited a higher stiffness, resulting in minimal deformation of the PDMS surface. In these cases, the EG droplets maintained a stable position and did not undergo significant structural changes, as evidenced by the flat or slightly concave geometry of the surface after curing. Conversely, at lower curing agent ratios (e.g., 20:1 or 40:1), the increased elasticity of the PDMS substrate enabled greater deformation under the weight of the droplets, resulting in pronounced negative deformation (concavity) beneath the droplets (Figure 2).

The observed deformation was attributed to the interplay between the mechanical properties of the PDMS and the evaporation of water. At lower curing agent ratios, the elastic properties of PDMS improve surface flexibility, enabling the droplets to exert sufficient pressure to create a concave shape; however, at excessively low curing agent ratios (e.g., above 40:1), the substrate becomes overly flexible and is therefore unable to maintain a stable concave geometry during curing. Accordingly, the z-axis position of the EG droplets and the resulting concave structure are governed by the viscoelastic properties of PDMS, which can be tailored by adjusting the mixture ratio. Thus, the optimal range of PDMS mixture ratios must balance elasticity and structural integrity to ensure the formation of uniform concave microwells. This tunability offers a simple yet effective approach for controlling the morphology of PDMS microwells, providing flexibility for various biomedical applications requiring precise structural features [15,16].

A PDMS ratio of 20:1 was used for microwell fabrication in this study. This ratio provides a more flexible and elastic surface than other PDMS mixtures, including the common 10:1 ratio. This 20:1 mixture affords increased elasticity that enhances the water vapor permeability of the material while maintaining its structural integrity. Softer PDMS matrixes allowed the formation of smooth microwell edges as the droplets contracted during the water evaporation process, forming a concave structure with a smooth-sloped profile. The elasticity of the PDMS is critical in preventing cracking or deformation during microwell formation, which is a common challenge for rigid materials. In addition to gas permeability, the shape and formation of the microwells are affected by the surface properties of PDMS, including its hydrophobicity. Water droplets exhibit a high contact angle on the hydrophobic surface of PDMS, which contributes to the smooth-sloped concave geometry of the microwells [17]. As water evaporates, the EG component within the preformed droplets is left behind, resulting in the formation of concave microwells with well-defined and reproducible geometries that can be controlled by simply adjusting the droplet volume and curing conditions. Microwell fabrication via water vapor permeation through PDMS highlights the importance of the gas permeability, elasticity, and surface characteristics of the material. By optimizing the fabrication process, smooth-sloped concave microwells were obtained using a PDMS ratio of 20:1.

### 3.2. Impact of Droplet Volume on Microwell Formation Yield

**Figure 3:**
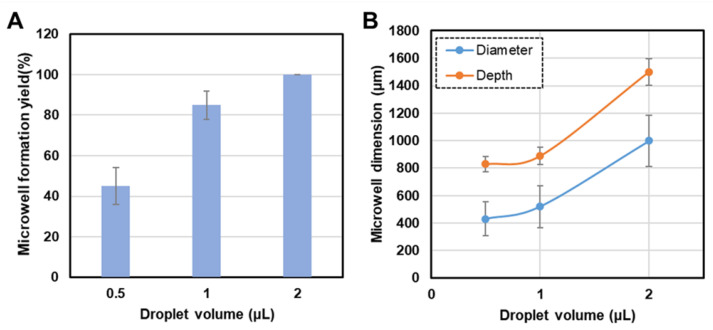
Effect of droplet volume on the microwell formation yield and final microwell dimensions. (**A**) Microwell formation yield (%) as a function of EG droplet volume (0.5 µL, 1.0 µL, and 2.0 µL) on 20:1 PDMS. (**B**) Microwell dimensions (diameter and depth) in 10:1 PDMS after replication using the mushroom-shaped micropillar mold, measured using different initial droplet volumes.

The volume of the 10% EG droplets deposited on the PDMS substrate is critical in determining the overall microwell formation yield. Therefore, the droplet volume was systematically varied to investigate its effect on the formation efficiency of concave microwells. A clear correlation between the droplet volume and microwell formation yield was observed, with the formation efficiency increasing with droplet volume (Figure 3A). The microwell formation yield, expressed as a percentage, is defined as the number of successfully formed microwells relative to the total number of droplets on the PDMS substrate. Droplets with volumes of 0.5 µL, 1.0 µL, and 2.0 µL were tested to evaluate the impact of droplet volume on the formation yield. A notable increase in microwell formation yield was observed with increasing droplet volume, with the 2.0 µL droplets producing the highest yield.

This trend can be explained by the contraction of the droplets during the evaporation of water. Smaller droplets (e.g., 0.5 µL) undergo less pronounced contraction owing to their limited volume of water, resulting in lower microwell formation efficiency. A lower water volume may result in incomplete contraction, thereby forming irregular or incomplete microwells. Furthermore, smaller droplets may increase the probability of premature drying or deformation before a microwell is fully formed. By contrast, larger droplets contain more water and therefore undergo more significant contraction during evaporation. As water vapor escapes through the PDMS membrane, the remaining EG undergoes a more uniform contraction to form well-defined microwells. A larger volume of water also ensures that evaporation occurs more slowly, allowing for the consistent, controlled, and stable formation of well-structured microwells, resulting in higher formation yields.

Additionally, larger droplets generated microwells with more uniform geometries, owing to the increased volume of EG remaining after water evaporation. This excess EG maintains the structural integrity of the microwell and prevents its collapse during the curing process. By contrast, smaller droplets may not contain sufficient EG to maintain the microwell structure, resulting in lower yields and less consistent geometries.

Although larger droplet volumes (e.g., 2.0 µL) continue to increase the overall microwell size, the formation yield plateaus at higher droplet volumes because the impact of volume on the microwell creation process is limited. Upon reaching a critical droplet size, further increases in volume primarily affected the dimensions of the microwell (diameter and depth) rather than its formation efficiency, suggesting that microwell formation yield can be effectively optimized by adjusting the droplet volume. An appropriate droplet size could maximize the formation efficiency and tailor the dimensions of the microwells for specific applications. We found that a droplet volume of 2 μL provided a good balance between formation yield and microwell uniformity and is therefore an ideal choice for applications requiring larger or deeper microwells.

### 3.3. Microwell Replication Using SU-8 Mold and Dimensional Characterization

The smooth-sloped concave microwells were replicated using an SU-8 mold, and the dimensional characteristics of the microwells replicates was evaluated. An SU-8 photoresist was selected as the molding material because of its high resolution and stability during microfabrication. The SU-8 molds enabled the accurate replication of microwell geometries, preserving the concave structure with high fidelity. The initial step in the replication process is the formation of a mushroom-shaped micropillar mold using SU-8 photoresist. The mold was polymerized over the original microwells created on a 20:1 PDMS substrate using UV irradiation. The low structural stability of the microwells formed directly on the 20:1 PDMS substrate necessitated the replication of the microwells using SU-8 models. Although the PDMS microwells exhibited smooth-sloped concave structures (Figure 1C), significant deformation occurred during mechanical manipulation, such as peeling or handling. The dotted yellow line in the optical image shows the interface where the PDMS base adhered to the surface of the petri dish, illustrating the structural deformation that occurs during mechanical manipulation. This instability was a consequence of the high elasticity of the 20:1 PDMS substrate, which lacked the mechanical rigidity required to maintain the integrity of the concave microwell structures under stress. To overcome this limitation, robust and dimensionally stable templates consisting of SU-8 molds were fabricated to ensure accurate replication of microwell geometries. The SU-8 mold also ensures high fidelity in preserving the smooth concave morphology and facilitates applications that require high mechanical stability.

**Figure 4:**
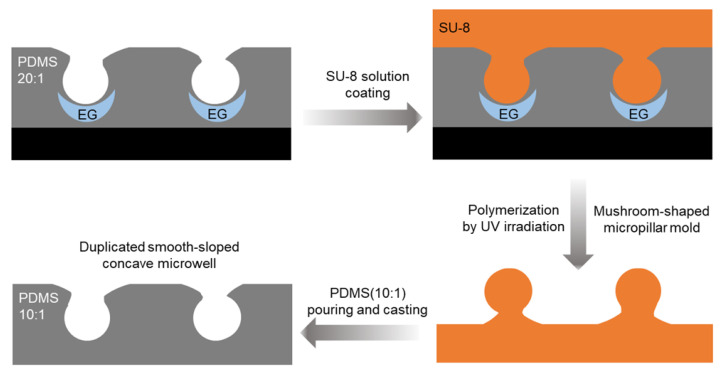
Fabrication of smooth-sloped concave microwells using an SU-8 mold and PDMS casting. A smooth-sloped concave microwell was initially formed using a PDMS mixture at a 20:1 ratio with embedded EG droplets. Subsequently, SU-8 photoresist was coated onto a microwell structure. Polymerization under UV irradiation formed a mushroom-shaped micropillar mold, which was used to cast a new microwell structure by pouring and curing a 10:1 PDMS mixture, thereby replicating a smooth-sloped concave microwell.

**Figure 5:**
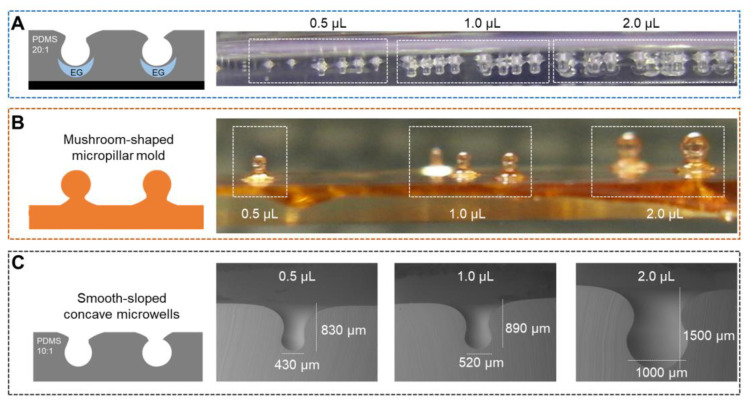
Optical microscopy and SEM images of concave microwell fabrication and corresponding mushroom-shaped micropillar molds. (**A**) Schematic and optical microscopic images of the concave microwells formed using EG droplets (0.5 µL, 1.0 µL, and 2.0 µL) on a PDMS (20:1) substrate. (**B**) Optical images of mushroom-shaped micropillar molds fabricated via UV polymerization of SU-8. The molds were created from the microwells formed by each EG droplet volume (0.5 µL, 1.0 µL, and 2.0 µL) shown in panel (A). (**C**) SEM images of the duplicated smooth-sloped concave microwells fabricated with PDMS (10:1) using the mushroom-shaped micropillar molds. The dimensions of the microwells formed using each EG droplet volume were measured.

The polymerization process ensured that the SU-8 mold precisely retained the structural features of the microwells, including a smooth-sloped entrance and a concave profile. The resulting mushroom-shaped micropillars were then used as negative molds to replicate the microwells during the subsequent PDMS casting. Thus, the 10:1 PDMS mixture, which provides a balance between mechanical strength and elasticity which ensures accurate replication and facilitates extraction from the mold, was poured onto the SU-8 mold and cured. After curing, the replicated microwells were removed from the SU-8 mold and the dimensions of the replicated structures were compared with those of the original microwells (Figure 4). The replicated PDMS (10:1) microwells retained the smooth-sloped concave structure of the original PDMS (20:1) microwells. The diameter and depth of the microwells, measured using SEM, were consistent with those of the original mold, demonstrating that the SU-8-based replication method produced high-fidelity replicas of the initial microwells (Figure 5B,C).

The use of the SU-8 mold enabled the replication of concave microwells with a high degree of structural fidelity and preserved the smooth-sloped profiles of the microwells, as confirmed by SEM (Figure 5C), which is critical for applications that require uniform cell growth or deposition of materials in a controlled environment, such as cell culture and drug testing platforms [11]. The replication process was also repeatable, allowing the production of multiple PDMS microwell arrays from the same SU-8 mold. This scalability enables the mass production of microwells without the need to repeatedly fabricate new molds and is one of the key advantages of the SU-8 mold. The SU-8 mold is also sufficiently robust to withstand multiple casting cycles without degradation or loss of dimensional accuracy [18,19].

Figure 3B shows the relationship between the initial droplet volume of EG and the dimensions (diameter and depth) of the microwells fabricated on 10:1 PDMS after replication. Both the diameter and depth of the microwells increased from approximately 400 μm to 1000 μm as the droplet volume increased from 0.5 µL to 2.0 µL. This significant increase reflects the growth of the contact area between the droplet and the PDMS substrate as the droplet volume increases. Similarly, the depth of the microwells increased from approximately 800 µm to 1500 µm with the same increase in droplet size. The increase in depth is correlated with the increased evaporation-driven deformation caused by the higher volume of the liquid and prolonged interaction with the substrate. These results underscore the tunability of the microwell dimensions by precisely controlling the droplet volume. The ability to modulate both the diameter and depth offers significant flexibility in fabricating microwells tailored specific applications such as cell culture and tissue engineering, in which the geometry of the microwell can directly impact biological outcomes. In addition, the use of SU-8 molds to replicate smooth-sloped concave microwells provides a reliable and scalable method for producing high-fidelity PDMS microwell arrays. Dimensional characterization confirmed that the replication process preserved the structures of the original microwells. Thus, this technique is suitable for applications requiring precise and consistent microscale features.

### 3.4. Cell Spheroid Formation on the Microwell Platform

The fabricated smooth-sloped concave microwells serve as an innovative platform for the formation of cell spheroids, which are critical components in 3D cell culture systems. Spheroids are clusters of cells grown in a 3D environment that more closely resembles the physiological conditions of tissues than conventional 2D monolayer cultures. The concave geometry of the microwells facilitates the formation of stable spheroids by promoting natural cell aggregation and enhancing cell–cell interactions.

**Figure 6:**
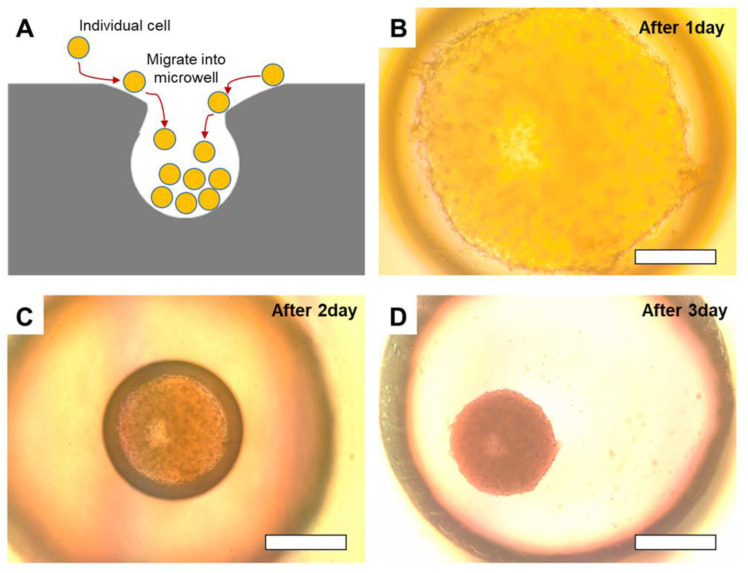
Formation of cell spheroids in smooth-sloped concave microwells over time. (**A**) Schematic showing individual cells migrating into the concave microwell via the smooth-sloped entrance, allowing efficient aggregation at the bottom of the microwell. (**B**–**D**) Optical microscopy images of spheroid formation at different time points. (**B**) After 1 day, cells begin to aggregate in the microwell. (**C**) After 2 days, a more compact and spherical structure begins to form. (**D**) After 3 days, a well-defined cell spheroid is observed. Scale bars: 300 μm.

To evaluate the efficacy of the microwell platform, microwells created using water vapor permeation were seeded with SW1222 cells. The cells migrated into the concave microwells over several days, gradually forming aggregates at the bottom of the microwell owing to the smooth-sloped entrance, which facilitated efficient cell migration. Optical microscopy observations at various times revealed the progression of spheroid formation: on day 1, the cells began clustering at the base of the microwell; by day 2, compact aggregates were formed; and by day 3, fully formed, well-defined spheroids were observed (Figure 6). This gradual formation process demonstrates the effectiveness of microwell geometry in supporting natural cell aggregation without external chemical or mechanical stimulation. The concave structure of the microwells facilitated cell entry and aggregation while maintaining spheroid integrity by preventing the premature dissociation of cells. This unique design mimics the physical constraints of in vivo conditions and provides a physiologically relevant 3D cell culture environment.

The ability of the microwell platform to control spheroid size by varying the dimensions of the microwells offers a significant advantage over existing methods. The diameter and depth of the microwells, which are influenced by the droplet volume during fabrication, directly affect the size of the spheroids. Larger microwells, produced using larger droplets (e.g., 2.0 µL), will produce larger spheroids, while smaller microwells (e.g., 0.5 µL droplets) will generate smaller spheroids. This tunability affords precise control over the dimensions of the spheroids, which is crucial for applications in which spheroid size influences biological outcomes. For example, limited oxygen and nutrient diffusion in the core of larger spheroids may create a hypoxic environment that mimics those of solid tumors, making larger spheroids suitable for tumor model studies [20,21]. Conversely, high-throughput drug screening and testing applications where uniformity and rapid growth are essential are better served by smaller spheroids [22,23].

The ability to reproducibly form cell spheroids in microwells developed in this study has significant implications for a range of biomedical and biotechnological applications. The spheroids generated in these microwells can serve as building blocks for larger tissue constructs, enabling the precise engineering of tissues with desired properties and thus providing a promising tool for regenerative medicine [4,24]. The platform is also highly suited to the creation of tumor spheroids that replicate the structure and microenvironment of solid tumors, facilitating the evaluation of anticancer drug penetration and efficacy under physiologically relevant conditions [2]. In addition, the microwell platform provides a scalable and efficient method for producing uniform muscle cell spheroids that can differentiate into muscle fibers with a consistent structure and quality, supporting the mass production of cultured meat [8,25]. Furthermore, microwells provide a controlled environment ideal for studying stem cell behavior and differentiation, enabling the growth of stem cell spheroids that offer insights into tissue regeneration and developmental biology [26].

The scalability and reproducibility of the microwell platform further enhance its utility. Multiple arrays of microwells with consistent dimensions were produced using the same SU-8 mold to ensure uniform spheroid formation. This consistency reduces the variability in experimental results and supports high-throughput applications such as drug screening or cultured meat production. The platform offers a versatile, scalable, and reproducible system for the formation of cell spheroids and provides new opportunities for biomedical and biotechnological innovation.

## 4. Conclusions

This study presents a novel and straightforward method for the fabrication of smooth-sloped concave microwells that relies on the unique water vapor permeability of PDMS. Microwells were formed through the controlled evaporation of water from EG droplets deposited on a PDMS substrate. The dimensions and yield of the microwells were strongly influenced by the initial droplet volume, with larger droplet volumes resulting in higher formation yields and larger microwells, thus demonstrating the tunability of the fabrication process. This method provides a simple, scalable, and lithography-free approach to create well-defined microwells for a wide range of applications. The production of mushroom-shaped micropillars via a mold to replicate microwells with different PDMS ratios (10:1) further demonstrated the versatility of the microwell platform. This replication process exhibited high fidelity and robustness, underscoring the potential of the platform for the scalable production of microstructures. The concave microwells fabricated in this study were highly effective 3D cell culture platforms that facilitated the natural aggregation of SW1222 cells and the formation of cell spheroids. The smooth-sloped design ensured efficient cell migration and aggregation, creating a controlled microenvironment that facilitated spheroid stability and growth. The potential applications of this platform extend beyond 3D cell culture and include tissue engineering, cancer research, and drug screening, which require precise control of the microenvironment. Furthermore, the microwell system shows promise for applications in the cultured meat industry, where uniform cell spheroids could serve as the basis for the large-scale production of muscle fibers. Future studies will optimize the fabrication process for the industrial-scale production of microwells and explore additional applications in regenerative medicine and biotechnology. To enhance the utility of the microwell platform, further studies will investigate the mechanical properties and biological behavior of the spheroids formed within these structures, including an evaluation of the relevance of spheroid size and geometry in specific biomedical applications such as drug efficacy testing and tissue regeneration. This versatile microwell platform, which exhibits exceptional scalability, reproducibility, and versatility, provides a valuable tool that will advance research and applications of 3D cell culture systems.

## Data Availability

Data will be made available on request.
